# High NT pro-BNP levels in children with malignant disorder receiving intensive fluid treatment: a prospective comparative study

**DOI:** 10.3389/fped.2024.1408231

**Published:** 2024-11-27

**Authors:** Weronika Pawlik, Joanna Strzemecka, Albert Stachura, Aleksandra Królak, Tomasz Ociepa

**Affiliations:** ^1^ Department of Pediatrics, Haemato-Oncology and Gastroenterology, Pomeranian Medical University, Szczecin, Poland; ^2^ Department of Methodology, Medical University of Warsaw, Warsaw, Poland

**Keywords:** ambulatory blood pressure monitoring, children, hematological malignancies, intensive fluid therapy, cardiac biomarkers, atrial natriuretic peptide (NT-proBNP), high-sensitivity troponin t (hs-TnT)

## Abstract

Hematologic malignancies are a well-known risk factor for cardiovascular disease development. Chemotherapeutic protocols commonly include intensive fluid therapy (IFT), which may negatively influence the cardiovascular system and predispose to arterial hypertension. This study aims to evaluate atrial natriuretic peptide (NT-proBNP), high-sensitivity troponin T (hs-TnT), and changes in blood pressure in children with hematological malignancies undergoing intensive fluid therapy. This prospective cohort study comprised thirteen children. 24-h ambulatory blood pressure monitoring (ABPM) and concentrations of NT-proBNP and hs-TnT were performed on the first day of IFT and during follow-up. There were no statistically significant differences in 24-h, daytime, night-time systolic (SBP) and diastolic blood pressure (DBP), SBP and DBP dipping, and the number of non-dippers during intensive fluid therapy compared to the control points. The mean NT-proBNP concentration at 24 h was 321.27 ± 318.08 pg/mL and was significantly higher compared with baseline (79.13 ± 105.42 pg/mL) and follow-up (175.92 ± 241.48 pg/mL); *p*-values 0.005 and *p* = 0.006 respectively. Troponin T concentration at 24 h was not significantly different compared with baseline and follow-up. These results show no significant influence of intensive fluid therapy on blood pressure profile. In contrast, an increase in NT-proBNP values 24 h after the start of fluid therapy may reflect the impact of fluid overload on the cardiovascular system.

## 1 Introduction

Elevated blood pressure (BP) and cardiac failure, as evidenced by studies, are common complications that occur after long-term anti-cancer therapy (1–5). The developing cardiovascular system in children is highly susceptible to the toxic effects of chemotherapy (e.g., anthracyclines), and damage of the blood vessel wall, including the endothelium (6, 7). The prevalence of atrial hypertension (AH) is significantly increased in a group of pediatric patients with hematopoietic and lymphatic malignancies (8). It is also well known that the rate of AH in childhood acute lymphoblastic leukemia (ALL) survivors is significantly higher than in the healthy pediatric population (9, 10).

Fluid overload and hypertension are related health conditions that often coexist, creating a complex interplay between fluid balance and blood pressure regulation. Fluid overload occurs when the body accumulates an excessive amount of extracellular fluid (11). This can result from various factors such as kidney dysfunction, heart failure, liver disease, or excessive fluid intake (12). In arterial hypertension, fluid overload contributes to an increased blood volume, exacerbating the pressure on arterial walls. Understanding the relationship between these two conditions is crucial for effective management and prevention of associated complications.

Therapeutic protocols used to treat children with leukemia or lymphoma include continuous coadministration of intravenous fluids. Numerous side effects of chemotherapy, mainly when cyclophosphamide, high-dose cytarabine, or high-dose methotrexate are given, may be minimized using intensive intravenous fluid therapy (13). Intravenous hydration of 3,000 mL/m^2^/day is usually recommended even several days after the drug administration, far exceeding a child's standard fluid requirement (14). However, the impact of such overhydration needs to be studied more, especially if it could increase the risk of cardiovascular complications. It is suggested that electrolyte disturbances and unrecognized fluid overload may negatively influence the cardiovascular system, predispose to arterial hypertension development, left ventricular hypertrophy and at last increases morbidity in patients with leukemia and lymphomas (15, 16).

A meta-analysis involving 7,507 children demonstrated an association between fluid overload and poorer outcomes of the underlying disease, worsening respiratory function, development of acute kidney injury, and mortality (17). Other observations also support an increased risk of developing respiratory adverse events in patients who are overhydrated during the induction phase of treatment for acute myeloid leukemia (18). What is more a study of patients with chronic kidney disease regularly undergoing hemodialysis demonstrated an association of chronic hypervolemia with blood pressure values and left ventricular hypertrophy (19–23). It appears that HSCT procedure in children who had a >10% overload of fluids during the initiation of hemodialysis increased the level of mortality (24). Conversely, some studies showed that even very aggressive hydration brings more positive outcomes than delay in methotrexate elimination (25–27).

For proper diagnosis and monitoring of AH, ambulatory blood pressure monitoring (ABPM) is currently considered the gold standard (28, 29). In children, a single blood pressure measurement has been shown to be significantly different from measurements obtained by ABPM in up to 40% of cases (30). ABPM, in addition to its ability to measure blood pressure almost continuously, allows assessment of blood pressure variability (BPV) throughout the day and night (31). Sustained increases in BPV are associated with increased incidence and progression of organ damage (renal dysfunction, left ventricular hypertrophy and cardiac dysfunction) (32–34).

Moreover, to assess the degree of myocardial overload or damage, methods include measurement of N-terminal natriuretic propeptide type B (NT-proBNP) and high-sensitivity troponin T (hs-TnT) (35). Changes in NT-pro BNP and hs-TnT may reflect the initial stage of cardiotoxicity even before the onset of signs suggestive of heart failure visible in imaging studies (echocardiography) (36, 37). NT-pro BNP levels have been shown to reflect left ventricular volume or pressure overload (38). Sommer et al. demonstrated the relationship between NT-proBNP levels and the degree of hydration in patients with heart disease and confirmed that an increased concentration of NT-proBNP is a factor in a poorer prognosis (39). It was also found that using NT-proBNP to monitor the hydration status of hemodialysis patients may have prognostic significance (40, 41). Highly sensitive troponin T (hs-TnT) is a marker of acute myocardial ischemia (42, 43). Increased troponin T levels and its association with an increased risk of cardiomyopathy have been demonstrated in children with ALL receiving anthracycline drugs (44). Higher hs-TnT levels and poorer left ventricular function have also been found in adults treated for childhood acute lymphoblastic leukemia (45).

This pilot study aimed to evaluate the impact of intensive intravenous fluid therapy on blood pressure values and biomarkers of myocardial function - atrial natriuretic peptide (NT-proBNP) and high-sensitivity troponin T (hs-TnT) in children with hematologic and lymphoid malignancies.

## 2 Material and methods

### 2.1 Study design

This study was conducted as a hospital-based single-arm longitudinal observational study and was granted the approval by the Bioethical Committee of Pomeranian Medical University in Szczecin, Poland (approval no. KB-0012/46/2021-A-3381). Informed consent was obtained from all parents or legal guardians and all children older than 13 years in compliance with the Declaration of Helsinki.

### 2.2 Setting

The analysis included the results of the NT-proBNP, hs-TnT concentrations and 24-h blood pressure monitoring (ABPM) performed on children aged 3–18 on the day when intensive fluid therapy was administered in parallel with cytostatic drugs. Patients included in the study were hospitalized in the Department of Pediatric, Hemato-Oncology and Gastroeneterology of Pomeranian Medical University in Szczecin, from December 2021 to May 2023 and met the inclusion criteria of the study. The study group served as a control group, and the control point was set weeks further in the therapy plan when children had not received intensive fluid therapy for at least 14 days. 24-h ABPM and blood sampling for NT-proBNP and hs-TnT concentrations measurements were performed again. Data were collected from December 2021 to May 2023.

### 2.3 Participants

The study group comprised 13 patients (five girls and eight boys, aged 3–18 years, mean 10.21 ± 5.51 years) with newly diagnosed leukemia or lymphoma, in whom intensive fluid therapy of 3 L/m^2^ of body surface/24 h was given to increase elimination and minimize cytostatics toxicity. Exclusion criteria were as follows: age above 18 years, diagnosed arterial hypertension, treatment with angiotensin-converting-enzyme (ACE) inhibitors or other antihypertensive drugs, a chronic or severe medical condition possibly resulting in the development of arterial hypertension (e.g., renal disorders, hyperthyroidism, congenital heart disease).

### 2.4 Variables and data sources

All patients underwent a complete physical examination with an assessment of weight, height, and body mass index (BMI). During fluid therapy, each patient had a fluid balance carried out. When the fluid balance exceeded 250 mL/m^2^, children were given a loop diuretic (Furosemide) at a dose of 0.5 mg/kg body weight.

Blood samples for NT-proBNP and hs-TnT concentrations were collected on admission and 24 h after the start of intensive fluid treatment. Pediatric reference intervals and cut-off points for NT-proBNP and hs-cTnT were determined in a study on a large group of healthy children without coexisting clinical indications (46). For children aged one to nine, the upper limit (>90%) was set at 178 ng/L for NT-proBNP and 9 ng/L for troponin T concentrations. These cut-off points were used in our study.

Blood pressure assessments were performed using Spacelabs ABP model 90227 OnTrak, the ambulatory blood pressure monitor (Spacelabs Healthcare Ltd) validated for children. An appropriately sized cuff was chosen out of three available sizes. The ABPM device was placed on the child's non-dominant arm two hours before intensive fluid therapy to allow the child to perform daily activities. Measurements were taken automatically every 20 min during the day (8:00 am–10:00 pm), and every 30 min during sleep (10:01 pm–7:59 am). The ABPM recording was considered acceptable when a minimum of correctly performed measurements was at least 70% - a minimum of 20 readings during the day and 7 at rest. Blood pressure values were recorded during the 24 h (daytime and night-time, respectively) as systolic, diastolic, and mean arterial blood pressure (MAP).

Blood pressure (BP) in children is significantly affected by both gender and age. While reference values for ambulatory blood pressure monitoring (ABPM) in pediatrics exist, using them in standard statistical procedures can be challenging due to the uneven distribution of BP in children. Reference values were established by Wuhl et al. based on ABPM values in 949 healthy children and adolescents aged 5–20 years (47). ABPM values (systolic, diastolic and mean arterial pressure; MAP) at Wuhl et al. work were recalculated and expressed as 24h-SDS (standard deviation score), day-time-SDS as well as night-time-SDS using the LMS (least mean squares) formula for SDS analysis: SDS = {[Y/M(t)]L(t)-1}/[L(t) × S(t)] where Y is the child's individual arterial blood pressure (systolic, diastolic or mean), M(t) is median of Y, L(t) is the measure of skewness and S(t) is the coefficient of variation (47, 48).

Statement from the American Heart Association confirms that the ABP values in the Wühl dataset remain the best available reference (49). Kliknij lub naciśnij tutaj, aby wprowadzić tekst. Due to the small sample size of this study, we compare the ABPM results of our patients based on the reference values created by Wuhl et al. standardizing them using the LMS method.

### 2.5 Statistical methods

All continuous variables were presented as means ± standard deviations unless otherwise indicated. Categorical variables were presented as absolute numbers and percentages. Due to the small number of participants at the preliminary stage of the study, non-parametric tests were used. For continuous variables (NT-proBNP, hs-TnT concentrations), the Kruskal-Walli's test was used to compare outcome measures between the studied time points. In case of significant between-group differences, a *post hoc* paired Wilcoxon test was used for pairwise comparisons. The student's *t*-test was used to compare the means of measured blood pressure values. The Wilcoxon signed-rank test was used to analyze non-parametric paired data (SD score of ambulatory blood pressure measurement). The threshold for statistical significance was assumed at a *p*-value below 0.05. For multiple pairwise comparisons, the Bonferroni correction was applied. In the case of three between-group comparisons, the statistical significance threshold was lowered to 0.0167.

## 3 Results

Thirteen children under treatment were enrolled in the study. The mean age of the study group was 10.21 ± 5.51 years (range 3–18), and there was a predominance of males (62.0%). The mean height was 1.4 ± 0.32 meters (range 1–1.79), and the mean BMI was 17.89 ± 4.25 (range 13.11–27.26). Only one child was obese (BMI above 97 percentile), and two of the patients were underweight (BMI under 3 percentile). Eight (62.0%) of the patients had acute lymphoblastic leukemia, three (23.0%) patients had non-Hodgkin lymphoma (NHL), and two (15.0%) patients had Hodgkin lymphoma (HL). Patients’ characteristics are presented in Table 1.

**Table 1:** Patient's characteristics.

Clinical value			Intensive fluid therapy	Minimal value	Maximal value
Age (years)			10.21 ± 5.51	3.58	17.58
Sex	Male	8 (62%)			
	Female	5 (38%)			
Height (m)			1.4 ± 0.32	1.00	1.79
BMI (kg/m^2^)			17.89 ± 4.25	13.11	27.26
Primary diagnosis	Acute lymphoblastic leukemia	8 (62%)			
	Non-Hodgkin lymphoma	3 (23%)			
	Hodgkin lymphoma	2 (15%)			

There were no statistically significant differences in 24-h, daytime, night-time systolic (SBP) and diastolic blood pressure (DBP), MAP, and HR recorded during intensive fluid therapy compared to the control point. There were no significant differences in SBP and DBP dipping and the number of non-dippers during IFT compared to the control point. These data are presented in Table 2. There were also no significant differences in 24-h MAP, HR, systolic, and diastolic blood pressure SDS during IFT compared to the control point. These data are presented in Table 3.

**Table 2:** Comparison of ambulatory blood pressure monitoring results during intensive fluid therapy and in control point.

	IFT^a^ Mean ± SD	IFT Median	IFT Min. values	IFT Max. values	CP^a^ Mean ± SD	CP Median	CP Min. values	CP Max. values	*p*-value
SBP 24 h (mmHg)	103.31 ± 9.27	103	88	116	104.62 ± 11.11	106	84	124	0.3508
DBP 24 h (mmHg)	66.23 ± 7.29	65	50	74	68.69 ± 6.17	69	57	79	0.1561
MAP 24 h (mmHg)	78 ± 6.51	79	62	85	79 ± 7.21	78	65	89	0.3260
HR 24 h (mmHg)	98.75 ± 16.92	95	78	135	98.46 ± 16.24	96	73	115	0.1820
SBP day (mmHg)	105.54 ± 8.86	107	89	116	107.62 ± 11.27	108	85	126	0.2758
DBP day (mmHg)	67.92 ± 7.25	69	51	78	70.08 ± 7.92	68	58	84	0.2260
SBP night (mmHg)	97.15 ± 11.14	96	76	116	98.62 ± 9.53	98	82	116	0.3174
DBP night (mmHg)	61.31 ± 8.91	64	48	73	63.15 ± 4.81	63	54	72	0.2377
SBP dipping (%)	8.04 ± 6.37	7.17	−0.14	23.02	8.24 ± 5.25	9	−1.45	15.95	0.4411
DBP dipping (%)	9.63 ± 8.90	6.48	−0.57	30.55	10.58 ± 6.37	7.4	2.36	23.99	0.3447

^a^ IFT, intensive fluid therapy.

^b^ CP, control point.

**Table 3:** Standard deviation scores of ABPM.

Blood pressure	Intensive fluid therapy	Control point	*p*-value
24 h Systolic SDS (mmHg)	−1.22 ± 1.07	−1.20 ± 1.99	0.9063
24 h Diastolic SDS (mmHg)	−0.16 ± 1.53	0.34 ± 1.19	0.367
24 h MAP SDS (mmHg)	−0.51 ± 1.26	−0.31 ± 1.23	0.5417
24 h HR SDS (mmHg)	1.53 ± 1.55	1.14 ± 1.05	0.6101

**Figure 1:**
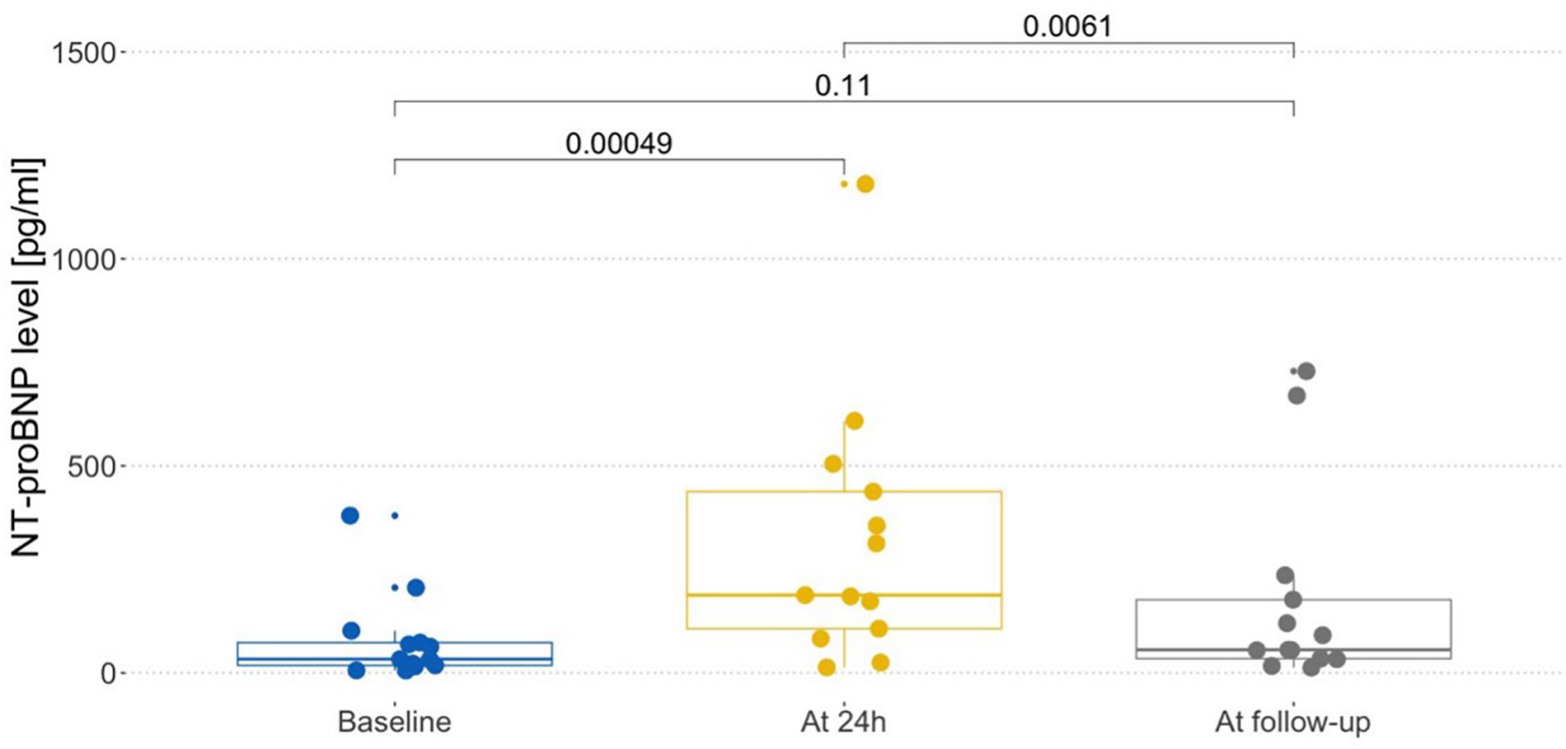
NTproBNP level in different time points.

**Figure 2:**
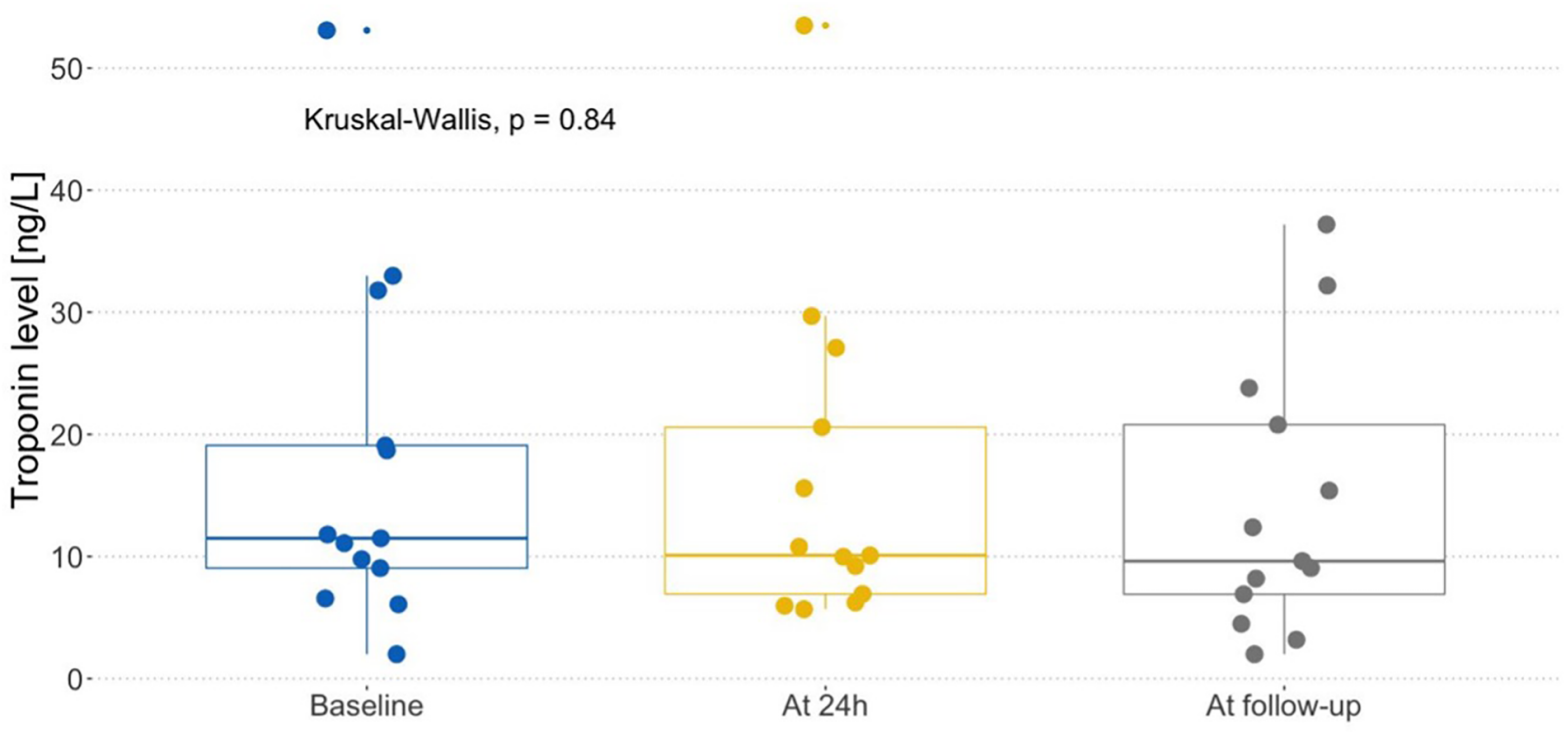
Troponin T level in different time points.

NT-proBNP concentrations differed significantly between time points (*p* = 0.022); therefore, post-hoc pairwise analyses were performed. The mean NT-proBNP concentration at 24 h was 321.27 ± 318.08 pg/mL and was significantly higher compared with baseline (79.13 ± 105.42 pg/mL) and follow-up (175.92 ± 241.48 pg/mL); *p*-values 0.005 and *p* = 0.006 respectively. The median NT-proBNP concentration at baseline was 33.4 pg/mL (range: 5.92–380 pg/mL), at 24 h was 188 pg/mL (range 13, 4–1,181 pg/mL) and at follow-up was 56 pg/mL (range: 12.9–729 pg/mL). These data are presented in Figure 1. Troponin T concentration at 24 h was 16.26 ± 14.13 ng/L and was not significantly different compared with baseline (18.47 ± 14.13 ng/L) and follow-up (15.27 ± 11 ng/L); *p* = 0.84. The median Troponin-T concentration at baseline was 11.5 ng/L (range: 3–53.1 ng/L), at 24 h was 10.1 ng/L (range 6.69–53.5 ng/L) and at follow-up was 9.63 pg/mL (range: 3–37.2 ng/L). These data are presented in Figure 2.

## 4 Discussion

Pediatric patients treated for leukemia and lymphomas are seven times more likely to die from complications, compared to healthy individuals (50). Furthermore, childhood cancer survivors have a higher risk of developing heart failure, coronary heart disease, heart valve insufficiency or stenosis, and inflammation of the heart epithelium after intensive systemic treatment, than non-cancer survivors (51–54). The burden on the heart is multifactorial, caused by various components of anticancer treatment, such as steroids, anthracycline chemotherapy, asparaginase administration, and concomitant fluids overload (51, 55). In severe sepsis, fluid boluses increase arterial blood pressure and lead to a significant decrease in HR and significant increases in cardiac output (56). However, it all may lead to many late-onset comorbidities (57).

Despite the routine use of IFT in children with hematologic and lymphoid malignancies at various stages of chemotherapy, its direct effect on the cardiovascular system, including blood pressure and its profile, as well as cardiac biomarkers, has yet to be studied. The standard is careful monitoring of the patient's water balance, control of renal and electrolyte parameters, and occasional (with varying frequency) blood pressure measurement. However, these procedures do not elucidate the direct effect of intensive fluid therapy on the cardiovascular system in children with hematological malignancies.

Some published papers confirm an increased prevalence of hypertension detected by ABPM in children with hematological malignancies a few years after diagnosis (4, 5, 58, 59). Moreover, Kumar et al. showed impaired nocturnal dipping, which may be evidence of an increased risk for cardiovascular ischemic events in children surviving acute lymphoblastic leukemia (59). Conversely, Hsiao W. et al. showed a significant reduction in median blood pressure loads many years after first diagnosis, which may reflect the transient nature of abnormalities in blood pressure parameters after the treatment termination (58).

This study is one of the few showing the profile of ambulatory 24-h blood pressure measurement in patients treated for hematologic malignancy, but to the best of the author's knowledge, the first one that aims to analyze diurnal changes in blood pressure in children undergoing intensive fluid therapy.

Our ABPM results show no statistically significant differences between the group of patients intensively hydrated and the controls. It was logical to expect that recorded ABPM values (means and SDS) should be higher during IFT than at the control point. However, we found no significant differences in 24-h, daytime, night-time systolic (SBP) and diastolic (DBP) blood pressure, MAP, and HR recorded during intensive fluid therapy compared to the control point. We also found no significant differences in 24-h MAP, HR, systolic, and diastolic blood pressure SDS in the same time points. True is that each patient received a loop diuretic during intensive hydration when the fluid balance exceeded 250 mL/m^2^. It needs further analysis if it would influence the values of recorded blood pressure in the patient's cohort. Small study groups may significantly impact results in both groups of patients. Although intensive fluid treatment continued during the night, we found no significant difference in SBP and DBP dipping and the number of non-dippers during IFT compared to the control point. Fluid overload and cardiac biomarkers are intricately linked, providing valuable insights into the cardiovascular status of patients (60). In fluid overload, the heart releases more NT-proBNP to counteract the increased workload. Troponins are elevated in conditions causing myocardial strain, including heart failure. Compromised cardiac function can lead to myocardial injury, triggering an increase in troponin levels.

So far, N-terminal pro-brain natriuretic peptide has been used as a prognostic marker for response to intensive chemotherapy and overall survival of patients with hematological malignancies (61–64). However, in the context of heart failure, NT-proBNP and troponin T was usually investigated to assess the development of anthracycline-induced cardiomyopathy (3, 36, 45, 65). This study investigated changes in cardiac biomarkers during excessive fluid supply and post-treatment follow-up, separately of the supply of drugs directly causing cardiotoxicity.

Our study showed a statistically significant increase in NT-proBNP concentration after 24 h of intensive fluid treatment (*p* < 0.005). Moreover, mean NT-proBNP concentration at this time was well above the upper limit (321.27 ± 318.08 pg/mL) established for the studied age group. It is also worth mentioning that the NT-proBNP levels had normalized by follow-up. Significant increase of NT-proBNP after 24-h of fluid therapy reflect the expected left ventricular and pressure overload, although it proved to be temporary.

The mean pre-IFT troponin T level was not significantly different from the concentration recorded after 24 h of intensive fluid treatment (*p* = 0.84).

It was a single-center, prospective study with a relatively small sample size, which limits generalizability. It is necessary to conduct the survey for several consecutive years to capture enough patients. For this reason, we conducted a preliminary study, which was necessary to minimize the risk of errors and adjust the methodology with subsequent patients.

These results may confirm only the transient influence of intensive fluid therapy on myocardial overload associated with acute neurohumoral activation (temporary elevation of NT-proBNP) indicating acute subclinical cardiotoxicity. If repeated or prolonged IFT significantly impairs left ventricular ejection fraction (LVEF) in children receiving intensive fluid treatment, it still needs to be assessed. Taking all these data into consideration, we speculate that IFT, if properly monitored and managed, has minimal impact on blood pressure and dipping status in children with a hematopoietic or lymphatic malignant disorder. Intensive fluid therapy should not be restricted, so that many of the adverse effects of cytostatic drugs can be avoided.

Although intensive fluid administration given concomitantly to anticancer treatment in children with hematological malignant conditions seems not to impact blood pressure negatively, an increase in NT-proBNP values 24 h after fluid therapy may reflect the level of fluid overload. If it will lead to further myocardial damage, a larger group of patients and longer follow-ups are needed to verify the accuracy of our findings. This study provides a prelude to further research into the negative effects of intensive therapy during chemotherapy.

## Data Availability

The raw data supporting the conclusions of this article will be made available by the authors, without undue reservation.
